# Editorial: Combining Simulations, Theory, and Experiments into Multiscale Models of Biological Events

**DOI:** 10.3389/fmolb.2021.797754

**Published:** 2021-11-19

**Authors:** Fabio Trovato, Joanna Trylska, Peter J. Bond, Peter G. Wolynes

**Affiliations:** ^1^ Department of Physics, Freie Universität, Berlin, Germany; ^2^ Centre of New Technologies, University of Warsaw, Warsaw, Poland; ^3^ Bioinformatics Institute (A*STAR), Singapore, Singapore; ^4^ Department of Biological Sciences, National University of Singapore, Singapore, Singapore; ^5^ Center for Theoretical Biological Physics, Rice University, Houston, TX, United States

**Keywords:** multiscale modeling, integrative modeling, molecular simulations, chromosome, binding, folding, multi-domain proteins, experiments

The number of publications that combine experiments and computer simulations has been growing steadily in the last 10 years. However, several challenges still need to be addressed in order to achieve a systematic integration, especially in the context of multiscale modeling of biological events. Computationally connecting the different scales—or more precisely the different system resolutions—constitutes one such difficulty of multiscale models. A second challenge is to choose the sources of experimental data that are most informative for parameterizing both the structural model and its inter-molecular interactions. This includes the modeling of the experimental conditions in order to avoid the misinterpretation of the results.

Quantum methods (QM) coupled to atomistic models (AT) have been instrumental in addressing, for example, the catalysis within an enzyme binding pocket. Five contributions in this special issue investigate biophysical events using multiscale QM/AT methods. To computationally explore slower events occurring in larger systems, it is often necessary to simplify the structural details of the system, an operation known as coarse-graining (CG). In this special issue, seven contributions employ such reduced models and three couple them with higher-resolution models, in a multiscale fashion. The interested reader is referred to the comprehensive reviews of Sun et al., and Giulini et al., as a guide to understanding the mathematical foundations of coarse-graining and multiscale methods, how to parameterize the inter-molecular forces and the relationship between a model resolution and its ability to reproduce the physicochemical properties of interest.

In this special issue, we have collected 22 manuscripts that emphasize the current efforts of the international scientific community to combine together experiments and computer modeling of biological events at multiple scales. A number of experiments have been performed across the different studies. They are cryo-EM, cryo-ET, FRET, EPR, AFM, CD, 3C^1^ and biological assays. Computational methods include MD, MC, NMA, MSM, ChK^2^, in silico mutations and whole-cell modeling. The investigated systems are represented at one or multiple among the QM, AT or CG resolutions or as a continuum, as in the Finite Element (FE) method.

Two research groups benchmark their methods for generating biomolecular structures. Fuchigami et al. combine AFM with MD simulations of a CG nucleosome model, modified to account for the AFM tip. With the aim of finding the nucleosome structure consistent with AFM images, the authors simulate nucleosome conformers while simultaneously inferring the geometric AFM tip radius on which the model (and the images) depend.

Kulik et al. combine a multiscale AT/CG protocol and mid-resolution cryo-EM maps to search the conformational space of multi-domain proteins and protein complexes sizing up to 2,211 residues. Their protocol is able to fit a flexible molecular model to cryo-EM maps with reasonable accuracy, therefore capturing states of a biomolecule that differ by large-scale movements, such as in the case of the DNA polymerase. The authors observe that their multiscale protocol is more beneficial for large biomolecules.

Harastani et al. present a cryo-ET sub-tomogram data analysis approach, called HEMNMA-3D, to study continuous conformational variability of the nucleosome. By generating a reference structure from the sub-tomograms and its normal modes, the authors retrieve known collective modes of a nucleosome. At variance with NMA-based methods, which fit an atomic structure to a cryo-EM map, HEMNMA-3D is designed to obtain a low-dimensional representation of the heterogeneity of a given set of EM maps, such as sub-tomograms.

Gilbert et al. devise a whole-cell modeling strategy and apply it to generate single-cell chromosome conformations of a genetically minimal bacterial cell, called Syn3A. Cryo-ET images are used to determine the cell size and ribosome distribution, within which the circular CG chromosome is embedded. This work is interesting in several aspects. A number of observations and model assumptions, grounded in the careful analysis of gene expression profiles and cryo-ET images, lead the authors to suggest that the chromosome of Syn3A cells has no substantial supercoiling or interactions between DNA segments that are distant in sequence. These initial assumptions are validated using contact maps calculated from preliminary 3C experiments and they generate a final cell model in which the circular chromosome is organized into territories and is not separated from the other cellular components, as instead occurs in *E. coli*. This study and that of Zha et al. emphasize that the generation of an accurate spatial model is often a delicate, preliminary step before any simulations can be conducted. In order to calculate the various model parameters, in fact, it is of utmost importance to choose informative biophysical and complementary methodologies to validate the computational results.

Several other DNA models exist in the literature, as reviewed by Sun et al.. Notable among them is oxDNA, which Sengar et al. describe in detail, discussing when it is worthwhile to use it and providing guidance to setting up and analyze the simulations.

Two additional large-scale models are presented by Zha et al., Saeedimasine et al.. The first research group tests an ultra coarse-graining strategy to generate microtubule models from 1 to 12 microns. Because these models reproduce known experimental mechanical properties, they are usable in future investigations aimed at understanding the supramolecular organization and small deformations of bundles of microtubules Saeedimasine et al.. propose a CG/FE multiscale model of the axolemma, which provides an explanation for the susceptibility of neurons to rupture under an applied mechanical stress. At the smallest scale, this model includes the myelin sheath and nodes of Ranvier lipid bilayers, represented at a CG resolution. Combining the results of the CG simulations with a FE model of the entire axon, the authors conclude that rupture is more likely to occur at the nodes of Ranvier instead of myelin, because myelin is observed to be more mechanically resistant.

Protein co-translational folding is the focus of the works of Chwastyk and Cieplak, and Yadav et al. The first research group studies how the geometry of the ribosome influences the co-translational folding of three model proteins, by performing simulations with different ribosome structures. The simulations suggest that protein synthesis is more obstructed in eukaryotes than in archaea and bacteria, which is consistent with constriction sites found in different positions of the exit tunnel of these ribosomes. Whether these constriction mechanisms are active in cells is unclear from the simulations alone because the ribosomes are approximated as rigid bodies. To predict the rate of protein synthesis or the populations of different nascent polypeptide states from an mRNA sequence, it is advisable to use mathematical and chemical kinetics modeling Yadav et al.. Since the predictions are sensitive to the codon translation rates used, Yadav et al. discuss how to estimate these kinetic parameters accurately from ribosome profiling data.

The characterization of intrinsic disorder in proteins is an important biophysical problem with many potential medical applications. An especially challenging aspect is the characterization of weak deviations from the random-coil state, due to protein residues that interact non-randomly and weakly. Towards this aim, Ritsch et al. combine distance distributions obtained from multiple EPR experiments with protein ensemble modeling. Because the method to analyze the raw EPR data typically influences the shape of the distance distributions used for structure generation, a model that attempts to detect weak deviations from a random-coil may contain a bias. To reduce such bias, the authors propose an overlap criterion for the distance distributions and a two-step modeling strategy to introduce the distance constraints. Under such controlled conditions, 19 distance distribution restraints are sufficient to observe deviations of a 133-residue segment from random-coil behavior.

Protein intrinsic disorder is also modulated by Post Translational Modifications (PTMs) and the presence of lipid membranes. Chiki et al. study the effects of PTMs on the aggregation of the first 17 amino acids of the Huntingtin protein. Key to the experimental success of their study is the production of phosphorylated T3 or acetylated K6 mutants within a background of oxidized M8. MD simulations suggest that the modified peptides explore a dynamic ensemble of disordered and helical conformations, with the helical conformations possessing a high fraction of short N-term helices. The increased content of N-terminal helical structure retards aggregation, in agreement with time-dependent AFM images. Consistent with a heterogeneous structural ensemble of the modified peptides, the distance distributions calculated from the simulations show a wide, often multi-peaked shape. Given the potential to detect weak deviations, Ritsch et al. EPR experiments performed on the Huntingtin protein could be used in the future to confirm these results.

Using molecular simulations, Christensen et al. study the effects of lipid bilayers of varying composition on the insertion mechanism of IAPP, a disordered peptide involved in glucose metabolism and type-II diabetes. The authors observe stabilization of IAPP helical states near the membrane and a dependence of IAPP insertion on the lipid composition. The presence of cholesterol causes the membrane to become more ordered and therefore less accessible to IAPP. The authors propose this mechanism to be protective with regard to amyloid formation.

Klein et al. extend their previous computational method to design a FRET sensor for the detection of cyclic guanosine 3′-5′-monophosphate (cGMP). As a first step, the authors generate the structure of the FRET sensor, which includes a linker and a FRET pair made of two fluorescent proteins. Subsequently, CG MD simulations are performed to exhaustively sample the conformations of the model sensor. The good agreement between the calculated and *in vitro* measured FRET efficiencies makes this method an interesting tool for the development of small molecule sensors.

Calmodulin (CaM) has been extensively investigated both computationally and experimentally. Nde et al. generate a CG model of CaM, which includes the effects of Ca^2+^ binding, albeit implicitly. The authors re-parameterize the AWSEM force field in order to reproduce CaM’s radius of gyration and the ratio of CaM’s open and closed states. Such a method might be applicable to study other flexible multi-domain proteins that respond to the binding of small molecules.

In the remainder of this editorial, we highlight six investigations on the theme of small molecule or protein binding to either proteins or RNA. Kaushik and Chang study the association between the HIV protease and xk263, under conditions of continuous flow of ligands close to a non-polar monolayer surface. Such conditions are reminiscent of those used in Surface Plasmon Resonance (SPR) for measuring binding constants. Besides the results of the Brownian dynamics simulations, this work could be applied to study the effects of solvent flow and surfaces on the association of any protein-ligand system, therefore paralleling and complementing SPR measurements.

Using enhanced MD simulations, Chyży et al. study the binding of neomycin to four single-mutants of the N1 riboswitch. The nucleobase mutations in the apical loop, although distant from the binding pocket, significantly affect the neomycin-riboswitch interactions. By carefully comparing their results with previous experimental work, the authors provide insights into the riboswitch structure-dynamics–activity relationship.

Narkhede et al. investigate the contribution of electrostatic forces to the selectivity of viral proteins SPICE and VCP towards the C3b complement protein, a key component of the complement immune system. By combining electrostatic calculations, in-silico alanine scanning and hotspot analysis, the authors identify sites resistant to local perturbation, where the electrostatic potential is likely to be evolutionary conserved. Their calculations are further supported by cofactor activity assays.

Liao et al. study the interactions between the neuropeptides PACAP and VIP with several receptors. Using a combination of homology modeling, enhanced MD simulations for the binding process and Markov State modeling, the authors find that different pathways are explored depending on the specific receptor/neuropeptide pair. The predicted receptor conformations and transition rates along the different binding pathways might be a useful dataset for guiding future drug design efforts.

Kalayan et al. investigate the influence of excipients on protein precipitation, which is relevant to the field of pharmaceutical protein formulations. The authors apply molecular simulations and their Energy-Entropy Multiscale Cell Correlation method to understand why lysozyme precipitates when interacting with the polyanion tripolyphosphate (TPP) but not with citrate. Overall, solvent and protein stabilization upon TPP binding are expected to drive binding via cross-linking with other proteins and therefore precipitation. By coarse-graining the current model, future simulations might enable observation of spontaneous cross-linking events, therefore validating this putative mechanism of precipitation.

Charzewski et al. propose a time-dependent QM/MM multiscale methodology to study the covalent docking between beta-lactamase and boron-based inhibitors. The authors apply this methodology to covalently dock three beta-lactamase enzymes and two boron-based inhibitors, obtaining several insights into the intermediate states. The simulation results suggest that this methodology could be effective in finding docking pathways by other boron inhibitors as well.

We conclude this editorial with a few additional messages. The first regards the importance of carefully estimating the experimental conditions. For example, when using FRET/EPR data, the effects of the fluorescent/spin label should be assessed either with control experiments Ritsch et al. or by directly incorporating the effects of the label into the model Klein et al. Similarly, the inclusion of geometrical features of an AFM tip can improve the model based on AFM images Fuchigami et al. The second message concerns the use of complementary experimental data when a single experimental technique is not sufficient Ritsch et al. or when it is necessary to validate a complex model Gilbert et al. Lastly, we underline that building a multiscale model is beneficial to test new hypotheses on complex events such as brain injury Saeedimasine et al. or to have access to multiple time and length scales for calculating different experimental quantities accurately Zha et al.

## Footnotes

1 MD, Molecular Dynamics; NMA, Normal Mode Analysis; MC, Monte Carlo; MSM, Markov State Modeling; ChK, Chemical Kinetics.

2 cryo-EM, Cryogenic Electron Microscopy; cryo-ET, Cryogenic Electron Tomography; FRET, Förster Resonance Energy Transfer; EPR, Electron Paramagnetic Resonance; AFM, Atomic Force Microscopy; CD, Circular Dichroism; 3C, Chromosome Conformation Capture.

